# Prenatal Air Pollution Exposure During Late Pregnancy Associates With Food Sensitization at 18 Months

**DOI:** 10.1111/cea.70340

**Published:** 2026-05-12

**Authors:** Edossa Merga Terefe, Richard Lundberg‐Ulfsdotter, Anna Oudin, Christian Asker, Magnus Domellöf, Christina E. West, Sophia Harlid

**Affiliations:** ^1^ Department of Diagnostics and Intervention, Oncology Umeå University Umeå Sweden; ^2^ Department of Clinical Sciences, Pediatrics Umeå University Umeå Sweden; ^3^ Department of Epidemiology and Global Health Umeå University Umeå Sweden; ^4^ Division of Occupational and Environmental Medicine, Department of Laboratory Medicine Lund University Lund Sweden; ^5^ Swedish Meteorological and Hydrological Institute (SMHI) Norrköping Sweden

**Keywords:** atopic dermatitis, DNA methylation, food allergy, NorthPop birth cohort, NO_x_, PM_2.5_, prenatal exposure, sensitization

## Abstract

- Air pollution exposure during development was associated with food sensitization (NO_x_) and atopic dermatitis (PM_2.5_).
- The associations were trimester‐specific, with evidence of effect modification by extrinsic and intrinsic factors.

To the Editor,

Prenatal exposure to air pollution is increasingly recognized as a potential contributor to early immune dysregulation [1] and the development of allergic disease in childhood [2]. Although substantial research has linked early‐life environmental exposures to adverse immunological outcomes, important uncertainties remain regarding the specific timing, mechanisms, and susceptibility factors that shape these relationships.

Using data from the NorthPop Birth Cohort Study (NorthPop), an ongoing population‐based study in Västerbotten County, Sweden (described in [3]), we examined whether prenatal exposure to nitrogen oxides (NO_x_) and fine particulate matter (PM_2.5_) was associated with food sensitization at 18 months and atopic dermatitis (AD) and food allergy (FA) by 3 years of age. We also explored whether cord blood DNA methylation mediates these associations, and whether intrinsic or contextual characteristics modify them.

Our analyses included up to 4358 mother–child pairs for AD and FA, and 2468 pairs for food sensitization. Air pollution exposures were estimated using high‐resolution dispersion modelling [4] based on maternal residential addresses. To account for temporal variation in exposure distributions across pregnancy and distinguish between potentially relevant time periods for immune system development, the pollutant exposures were categorized into trimester‐specific quartiles. This allowed for comparison of relative exposure levels within each trimester; however, effect estimates across trimesters are not directly comparable due to differences in the quartile cut‐off points. Clinical outcomes were derived from both the Swedish National Patient Register [5] and parental questionnaires, and food sensitization was determined using allergen‐specific IgE levels obtained at 18 months. This combination of biomarker, register, and survey‐based outcome data allowed for a comprehensive evaluation of allergic manifestations across early childhood.

We observed that higher prenatal exposure to NO_x_, particularly during the second and third trimesters, was associated with an increased risk of food sensitization at 18 months (Table 1). These associations appeared strongest when comparing the second‐lowest exposure category (Q_2_) to the reference (Q_1_), rather than when comparing the highest exposure quartiles to Q_1_. For instance, during the second trimester, Q_2_ exposure levels corresponded to substantially higher relative risks of food sensitization, while both Q_3_ and Q_4_ also demonstrated elevated risks in the later trimesters. This pattern suggests that the exposure–outcome relationship may not be strictly linear and that even moderate increases in NO_x_ exposure may be relevant for fetal immune programming. In contrast, no associations were detected between prenatal NO_x_ exposure and either AD or FA by age 3.

**TABLE 1:** Associations between trimester‐specific prenatal exposure to NO_x_ and PM_2.5_ and the risk of childhood allergic outcomes.

Pollutant	Quartile	First trimester^a^		Second trimester^a^		Third trimester^a^	
		RR^b^	95% CI	RR^b^	95% CI	RR^b^	95% CI
*Food sensitization* ^c^							
NO_x_	Q_1_	1.00 (ref)	—	1.00 (ref)	—	1.00 (ref)	—
	Q_2_	1.05	0.79–1.38	**1.41**	**1.07–1.86**	1.28	0.97–1.71
	Q_3_	1.21	0.92–1.58	1.22	0.92–1.63	**1.37**	**1.04–1.82**
	Q_4_	1.17	0.89–1.55	**1.37**	**1.04–1.83**	**1.35**	**1.02–1.80**
PM_2.5_	Q_1_	1.00 (ref)	—	1.00 (ref)	—	1.00 (ref)	—
	Q_2_	1.07	0.81–1.42	1.01	0.78–1.32	0.99	0.75–1.30
	Q_3_	1.23	0.94–1.61	0.85	0.64–1.11	1.05	0.81–1.38
	Q_4_	1.14	0.87–1.51	0.93	0.71–1.22	1.01	0.77–1.33
*Atopic dermatitis* ^c^							
NO_x_	Q_1_	1.00 (ref)	—	1.00 (ref)	—	1.00 (ref)	—
	Q_2_	1.03	0.84–1.27	1.16	0.96–1.41	1.04	0.85–1.28
	Q_3_	1.13	0.92–1.38	0.96	0.78–1.18	1.07	0.87–1.31
	Q_4_	1.16	0.95–1.42	1.02	0.83–1.25	1.15	0.94–1.40
PM_2.5_	Q_1_	1.00 (ref)	—	1.00 (ref)	—	1.00 (ref)	—
	Q_2_	0.99	0.81–1.21	1.20	0.98–1.47	1.04	0.86–1.27
	Q_3_	0.92	0.75–1.13	**1.23**	**1.01–1.50**	0.99	0.82–1.21
	Q_4_	0.99	0.81–1.21	1.09	0.89–1.34	0.91	0.74–1.11
*Food allergy* ^c^							
NO_x_	Q_1_	1.00 (ref)	—	1.00 (ref)	—	1.00 (ref)	—
	Q_2_	1.11	0.80–1.54	0.96	0.68–1.34	1.00	0.73–1.39
	Q_3_	1.11	0.81–1.53	1.16	0.84–1.60	0.95	0.69–1.33
	Q_4_	0.95	0.68–1.33	1.02	0.73–1.41	1.00	0.73–1.39
PM_2.5_	Q_1_	1.00 (ref)	—	1.00 (ref)	—	1.00 (ref)	—
	Q_2_	1.24	0.89–1.73	0.89	0.64–1.23	0.87	0.63–1.18
	Q_3_	1.34	0.97–1.87	0.93	0.67–1.28	0.83	0.61–1.14
	Q_4_	1.13	0.80–1.59	1.00	0.73–1.37	0.75	0.54–1.03

*Note:* Bold values show a statistically significant estimate.

^a^ Each outcome was modelled separately based on each pollutant for the first, second, and third trimesters of pregnancy. Quartile cutoffs for NO_x_ and PM_2.5_ were based on trimester‐specific distributions.

^b^ Results are presented as relative ratios (RRs) with 95% confidence intervals (CIs) comparing higher quartiles of exposure (Q_2_–Q_4_) to the reference quartile (Q_1_).

^c^ The models were adjusted for the highest level of education, maternal age at delivery, maternal smoking during pregnancy, parental history of disease, parity, and pet exposure.

For PM_2.5_, we found no evidence that prenatal exposure increased the risk of food sensitization or FA. However, second‐trimester PM_2.5_ exposure was significantly associated with higher risks of an AD diagnosis by 3 years of age. This finding was supported across multiple statistical analyses, including continuous modelling of exposures, mutual pollutant adjustment, and spline‐based evaluations of potential non‐linearity.

To assess the robustness of our findings, we conducted an extensive set of sensitivity analyses. These included restricting outcomes to questionnaire‐based diagnoses, limiting analyses to complete cases, evaluating both pollutants simultaneously, exploring non‐linearity, considering trimester‐adjusted exposures, and examining the average exposure across the entire pregnancy. Across these models, the direction and magnitude of effect estimates were generally consistent, particularly for the associations between NO_x_ and food sensitization and between PM_2.5_ and AD. These results reinforce the robustness of the trimester‐specific findings and highlight the importance of exposure timing in early immune development.

We also examined potential effect modification by three characteristics: maternal exposure to the COVID‐19 pandemic during pregnancy, child sex, and parental history of allergic disease. Interactions revealed notable heterogeneity. For example, the associations between early pregnancy exposure to NO_x_ or PM_2.5_ and FA were stronger among girls than boys, consistent with previous findings that immune responses and susceptibility to pollutants may differ by sex [6]. Likewise, parental history of allergic disease amplified the risk associated with prenatal pollutant exposures for several outcomes, suggesting that genetic or familial predisposition may heighten vulnerability to air pollution. The COVID‐19 pandemic emerged as another important contextual factor. Associations between pollutant exposures and FA appeared weaker among children whose mothers were pregnant during the pandemic, possibly reflecting reduced mobility, altered exposure patterns, or shifts in healthcare use during this period [7]. Taken together, these findings emphasize that prenatal pollutant effects are not uniform across populations but are shaped by both biological and contextual modifiers.

To further explore biological mechanisms, we performed epigenome‐wide association studies (EWAS) using cord blood DNA methylation profiles in a subset of 499 children. Although several CpG sites showed nominal associations with prenatal NOx exposure or food sensitization, none survived correction for multiple testing. High‐dimensional mediation analyses similarly did not provide evidence that DNA methylation mediates the relationship between NO_x_ exposure and food sensitization. The absence of statistically significant findings of DNA methylation mediation should be interpreted in light of several methodological considerations. First, the relatively small subsample size with methylation data may have limited statistical power to detect modest mediation effects, particularly in the context of EWAS involving many CpG sites and stringent multiple‐testing correction [8]. Second, the biological effects of prenatal air pollution exposure on DNA methylation may be subtle and distributed across multiple loci in a genomic region, rather than concentrated at individual CpG sites [9], which reduces the likelihood of identifying single‐site associations that meet significance thresholds. It is also possible that prenatal air pollution influences allergic disease risk through alternative or complementary pathways, including inflammatory mechanisms and the immune system [2], which may not be fully mediated by detectable changes in DNA methylation at birth.

In summary, our findings indicate that higher prenatal exposure to NO_x_ during mid‐to‐late pregnancy is associated with food sensitization at 18 months, while increased second‐trimester PM_2.5_ exposure is associated with AD by age 3. These associations were trimester‐specific, context‐dependent, and displayed possible non‐linear patterns. We found no evidence of mediation through DNA methylation. The results suggest that even moderate levels of air pollution may influence early immune development, highlighting the importance of temporal exposure windows and individual susceptibility characteristics.

## Author Contributions

S.H. and C.E.W. contributed to study design; E.M.T., S.H., and C.E.W. contributed to conceptualization; E.M.T., S.H., and C.A. contributed to methodology; E.M.T., S.H., A.O., C.E.W., and M.D. contributed to interpretation of data; C.E.W. and M.D. contributed to ethics approval; S.H., C.E.W., and M.D. contributed to funding acquisition; R.L.‐U. contributed to data collection, extraction, and curation; E.M.T. contributed to statistical analysis and data visualization and wrote the first manuscript draft. All authors revised the manuscript and approved the final version.

## Funding

This work was supported by Västerbotten County Council, RV 832 441, RV 967 569. Umeå University. FORTE. Kempestiftelserna, JCSMK23‐0177. Swedish Asthma and Allergy Association, F2018‑0027. Swedish Research Council, 2019‑01187, 2018‐02642, 2021‐01637. Swedish Heart and Lung Foundation, 2020‑0473, 2018‐0641. Ekhaga Foundation, 2018‐40. FORMAS, 2021‑01098. Thermo Fisher Scientific/Phadia.

## Ethics Statement

The research project was approved by the Ethical Review Authority of Sweden (2014/224‐31, 2015‐175‐31 M, 2017‐99‐32 M, and 2017‐368‐32) and was conducted in accordance with the Declaration of Helsinki. Written and informed consent was obtained from both parents.

## Conflicts of Interest

The authors declare no conflicts of interest.

## Data Availability

Data described in the manuscript will be made available upon reasonable request, pending valid ethical approval and approval by the NorthPop steering committee. A comprehensive Supporting Information, including full methodological descriptions, additional results, and extended tables and figures, is available on the Open Science Framework (OSF) at https://osf.io/w84zv/files/6s7ey.
